# Polyurethane biodegradation by *Serratia* sp. HY-72 isolated from the intestine of the Asian mantis *Hierodula patellifera*

**DOI:** 10.3389/fmicb.2022.1005415

**Published:** 2022-12-19

**Authors:** Jong-Hoon Kim, Seung Hoon Choi, Min Gu Park, Dong Hwan Park, Kwang-Hee Son, Ho-Yong Park

**Affiliations:** ^1^Microbiome Convergence Research Center, Korea Research Institute of Bioscience and Biotechnology, Daejeon, Republic of Korea; ^2^Department of Agricultural Biotechnology, College of Agriculture & Life Science, Seoul National University, Seoul, Republic of Korea

**Keywords:** polyurethane, polyurethanase, biodegradation, insect-associated microbe, *Serratia*, plastic waste management

## Abstract

Polyurethane (PU), currently replacing existing synthetic materials worldwide, is a synthetic polymer derived from polyols, isocyanates, and a chain extender added by condensation reactions. PU wastes which are difficult to recycle, are commonly discarded in landfills and flow into ecosystems, thereby causing serious environmental problems. In recent years, insect-associated microbes have become a promising, eco-friendly strategy as an alternative to plastic recycling. This study aimed to evaluate the potential of *Serratia* sp. HY-72 strain isolated from the intestine of the Asian mantis (*Hierodula patellifera*) for PU degradation. The 65 kDa family I.3 lipase which degrades PU was identified and characterized, with a specific activity of 2,883 U mg^−1^. The bacterial filtrates and the recombinant lipase degraded Impranil (a colloidal polyester-PU dispersion, 100 g l^−1^) by 85.24 and 78.35% after 72 h incubation, respectively. Fourier transform infrared spectroscopy analysis revealed changes in Impranil functional groups, with decreased C=O functional group and aliphatic chain signals, and increased N-H bending with C-N stretching and C-O stretching. The current study also revealed that the HY-72 strain biodegraded the commercial PU foams (polyester- and polyether- PU) with 23.95 and 10.95% weight loss after 2 weeks, respectively with changes in surface morphology and structure such as cracks, roughness, and surface roughening. Altogether, this is one of the few studies reporting biodegradation of PU by the insect-associated microbe. These findings suggest that the insect-associated microbe could be a promising resource for biodegradation and recycling of plastic waste.

## 1. Introduction

Plastics are composed of chains comprising numerous organic subunit linked by dynamic covalent bonds and are widely used owing to their stability, ease of manufacturing, economic efficiency, and convenience (Alabi et al., 2019; Atanasova et al., 2021; Roy et al., 2021). Plastic production has grown exponentially, with a very large number of plastics produced since 1950 (Bilal et al., 2021). Plastic disposal systems, such as incineration, recycling, and landfills, are ineffective in plastic waste management (Seneviratne et al., 2006). Plastic decomposition is extremely slow because of the chemical structure of most plastics and poor degradation capabilities of natural decomposition processes. Furthermore, it generates small particles, known as microplastics, which are more likely to cause serious ecological and biological concerns. In particular, the accumulation of plastic waste continues to have harmful effects on the ecosystem (Barnes et al., 2009; Mukherjee et al., 2011; Osman et al., 2018; Amobonye et al., 2021). Hazardous contaminants are eluted from plastic waste, causing the biological accumulation of harmful chemicals in the food chain and leading to problems such as endocrine disorders and reduced species diversity (Liaqat et al., 2020).

Polyurethane (PU) is a type of plastic with increasing usage over the decades because of its diverse properties and is currently replacing existing synthetic materials (Liu et al., 2021). PU was discovered and produced by Dr. Otto Bayer in 1937. Since then, it has become ubiquitous in modern life and is extensively used in medicine and industries (Howard, 2002). PU has a high melting point and tensile strength, improving its durability (Bayer, 1947). It is a polymer derived from the condensation of polyisocyanates and polyols with carbamate ester bonds (-NHCOO-; Nakajima-Kambe et al., 1999) and is classified into four main types according to the polyol composition (Vargas-Suárez et al., 2019). PU is commonly discarded in landfills or incinerated for heat production owing to its highly complex polymer structure (Ignatyev et al., 2014; Utomo et al., 2020). The decomposition of PUs is slow and produces serious pollutants (Liu et al., 2021). Furthermore, improper incineration generates toxic gases such as carbon monoxide and hydrogen cyanide that negatively affect human health and ecosystems (McKenna and Hull, 2016; Alshehrei, 2017). Physical and chemical degradation are inefficient in eliminating PU waste. Therefore, the widespread and increasing use of PU in modern society makes biodegradation as important as manufacturing these plastics.

Since the 1960s, several studies have confirmed that microorganisms and their enzymes can degrade PUs (Schmidt et al., 2017; Magnin et al., 2020). These microorganisms either use PUs directly as nutrient sources or indirectly degrade them with several enzymes and metabolites. Esterases, lipases, proteases, and ureases produced by microorganisms can biodegrade polyester-PU (Nakajima-Kambe et al., 1999; Howard, 2002; Loredo-Treviño et al., 2012) and proteases and esterases can directly disrupt urethane binding sites (Loredo-Treviño et al., 2012). Among the hydrolysis enzymes, lipases derived from bacteria are considered more suitable for industrial environments due to their large-scale substrate specificity (Javed et al., 2018). Despite continuous research and notable success, PU-degrading enzymes remain a challenge and require extensive research. Therefore, there is a pressing need for scientific and technological advances in context of bacterial hydrolytic enzymes for PU biodegradation.

Insects have developed symbiotic interactions with numerous microorganisms to overcome environmental limitations (Sudakaran et al., 2015). Recently, the plastic-degrading capability of insect-associated microorganisms is drawing attention. Insect intestinal microbiota has evolved to produce effective degradative enzymes and utilize various substrates as nutrients to overcome environmental limitations (Sudakaran et al., 2015; Berasategui et al., 2016). They are more functional than degradative enzymes produced by microorganisms in the free-living state, and have the potential of industrial applications (Jang and Kikuchi, 2020). Previous studies have highlighted that microorganisms extracted from insect intestines can be cultured to break down plastics such as polyethene (PE) and polystyrene (PS; Yang et al., 2014, 2018). These microorganisms isolated from insects are a promising source of end-of-life solutions for plastics and have the advantage of not causing secondary pollution from plastic waste (Lee et al., 2020).

In this study, the potential PU-degrading bacterium, *Serratia* sp. strain HY-72, was isolated from the intestine of the Asian mantis (*Hierodula patellifera*). PU-biodegrading potential of the strain HY-72 was evaluated by comparing weight loss, scanning electron micrographs, and chemical composition. In addition, family I.3 lipases with PU-degrading activity were identified and evaluated from the strain HY-72.

## 2. Materials and methods

### 2.1. Materials

The commercial polyester urethane dispersion agent, Impranil DLN (Impranil; Bayer Materials Science, PA, United States), was purchased to evaluate PU biodegradation by the isolated strain. The two types of PU foams (polyester- and polyether-) were purchased from KPX Chemical Co. (Seoul, Republic of Korea). Each PU foam sample was cut into small pieces (polyester PU, 20 by 30 by 15 mm; polyether PU, 8 by 30 by 15 mm) and weighed (polyester PU, 250 mg; polyether PU, 150 mg). Three replicates of each type were soaked in 70% ethanol for 1 h and washed with sterile distilled water. Subsequently, the pieces were place in an oven at 50°C until the surface moisture was removed.

### 2.2. Isolation and identification of PU-degrading bacteria

Adult Asian mantis (*H*. *patellifera*) were collected from Bomoon Mountain (Daejeon, Republic of Korea) and transported to the laboratory. Insects were dissected to isolate bacterial strains using the method described by Heo et al. (2006) with minor modifications. The insects were immersed in 70% (v/v) ethanol for 1–2 min to remove the surface contaminants and then washed twice with sterile distilled water. Next, the digestive tracts were removed, and the intestinal contents were carefully recovered. The intestinal contents were diluted in phosphate-buffered saline (PBS; 0.8% NaCl, 0.02% KCl, 0.144% Na_2_HPO_4_, 0.024% KH_2_PO_4_, pH 7.4) and spread onto one-fifth strength of Reasoner’s 2A (R2A) agar (KisanBio, Seoul, Republic of Korea) containing 1% Impranil to isolate the PU-degrading gut bacteria. After incubation at 30°C for 2 days, a HY-72 bacterial strain, which only formed a translucent halo around areas of bacterial growth, was chosen for further experiments. The bacterial strain was isolated and stored at −70°C in R2A broth with 25% sterilized glycerol. Genomic DNA was extracted from the strain HY-72 and 16S rRNA was amplified by polymerase chain reaction (PCR) for its identification. The universal primers 27F (5′-AGA GTT TGA TCM TGG CTC A-3′) and 1492R (5’-TAC GGY TAC CTT GTT ACG ACT T-3′) were used. The sequence of the 16S rRNA gene was compared with those of type strains available in the EzBioCloud database (ChunLab Inc., Seoul, Republic of Korea) to find closely related species. Molecular phylogeny of 16S rRNA was inferred by the neighbor-joining method in MEGA X software (Kumar et al., 2018).

### 2.3. Extracellular enzymatic activity

The strain HY-72 was assessed for protease and lipase production using an agar plate assay as described by Bhagobaty and Joshi (2012), with minor modifications. The revived bacterial culture was streaked onto R2A agar containing a specific substrate and incubated at 30°C for 3 days. The ability to produce protease was qualitatively confirmed by observing transparent halo zones surrounding the colonies on the R2A agar containing 2% (w/v) skim milk. The extracellular lipase activity was qualitatively determined using R2A agar with 1% (w/v) Tween 80 and 0.01% (w/v) CaCl_2_ and confirmed by the formation of crimson dots around the colonies, which were further measured.

### 2.4. Identification and characterization of PU-degradable enzymes

For the recombinant production in *Escherichia coli* BL21 (DE3) of mature lipase having PU-degrading capability, the encoding gene was amplified using specific primers, SLPoly-F containing an NdeI restriction site at the 5′-end (5′-CGCCATATGATGGGAATCTTTAAT-3′) and SLPoly-R containing a HindIII restriction site at the 5′-end (5′-GGCAAGCTTTCAGGCCAGTAC-3′), using strain HY-72 genomic DNA as a template. PCR was performed using a thermal cycler (Takara, Kyoto, Japan), and the initial template denaturation was performed for 2 min at 94°C, followed by 35 cycles of 10 s at 98°C, 30 s at 57°C, and 1 min at 68°C. The PCR-amplified fragment was inserted into the pET-28a (+) vector (Novagen, Darmstadt, Germany) by a ligation reaction. After transformation of the ligation mixture into *E*. *coli* BL21 (DE3), the *E*. *coli* strain containing the recombinant plasmid was grown at 37°C in LB broth (BD Difco, Franklin Lakes, NJ, United States) supplemented with 50 μg ml^−1^ kanamycin. To overproduce lipase proteins, recombinant *E*. *coli* BL21 (DE3) cells harboring pET-28a (+)/lipase were cultivated in a 5-l baffled flask with 1 l of LB broth and 50 μg ml^−1^ of kanamycin in a rotary shaker (150 rpm) for 18 h at 30°C. Overexpression of the target gene was induced by adding 1 mM isopropyl *β*-D-1-thiogalactopyranoside when the optical density of the culture at 600 nm reached approximately 0.6. Following cultivation, lipase-expressing cells were harvested by centrifugation (10,000 × g) for 20 min at 4°C. The cells were thoroughly washed twice, re-suspended with PBS, and disrupted *via* sonication. Lysates were harvested by centrifugation (13,000 × g) for 10 min at 4°C. The recombinant protein in the lysate was purified using a Ni-NTA Fast Start Kit (QUIAGEN, Valencia, CA, United States) according to the manufacturer’s instructions.

The cleavage site for the protein’s signal peptide was predicted using the SignalIP 5.0 server.^1^ Conserved domain searches and protein family recognition were conducted using the Pfam server.^2^ The sequence of lipase from HY-72 was retrieved from the GenBank database by searching BLASTp.^3^ Multiple alignments of lipase amino acid sequences were achieved using Clustal W in MEGA X software. Phylogenetic analysis of lipase was conducted using MEGA X software with the neighbor-joining method. The reliability of the phylogenetic tree was checked by bootstrap analysis based on 1,000 replications.

The relative molecular mass of the purified lipase was analyzed using sodium dodecyl sulfate-polyacrylamide gel electrophoresis (SDS-PAGE; Mini-PROTEAN system, Bio-Rad, Hercules, CA, United States). After electrophoresis, the proteins separated by SDS-PAGE were visualized by staining the gel with Coomassie Brilliant Blue R 250, and zymopraphy was performed on the other side of the gel, according to Vandooren et al. (2013), with minor modifications. For zymograms, a gel containing purified recombinant lipase was washed with 50 mM Tris–HCl buffer (pH 8.0) for 15 min and subsequently placed on top of the zymogram gel with 1% Impranil as substrate. The zymogram was incubated overnight at 28°C and analyzed for clearing the following day.

Quantitative assays of protein concentrations were conducted using the Qubit 4 Fluorometer (Invitrogen, Thermo Fisher Scientific, Waltham, United States) and associated kit (Qubit Protein Assay Kit, Invitrogen, Thermo Fischer Scientific, Massachusetts, United States) according to the manufacturer’s instructions. The lipolytic activity of the purified enzymes was measured using a spectrophotometric method with *p*-nitrophenyl acetate (*p*NPA) as the substrate (Shin et al., 2021). The purified enzyme (0.1 ml) was added to 0.5 ml of 3 mM *p*NPA and 0.9 ml of 20 mM Tris-sulfate buffer (pH 7.5), and the biocatalytic reaction proceeded at 25°C for 5 min. After the reaction, the absorbance was measured using a UV/Vis spectrophotometer (DU 730^®^ Life Science UV/Vis spectrophotometer; Beckman Coulter, Brea, CA, United States) at 405 nm. One unit of lipase was defined as the amount of enzyme that hydrolyzed 1 μmol *p*-nitrophenol ml^−1^ min^−1^.

### 2.5. Impranil degradation assay

PS-PU Impranil biodegradation was quantitatively determined using cell-free supernatants of the strain HY-72 as described by Hung et al. (2016) with minor modifications. The isolate was cultured in a baffled Erlenmeyer flask containing 10 ml R2A medium on a rotary shaker at 30°C for 2 days. After incubation, the supernatants were separated by centrifugation at 10,000 × g and 4°C for 15 min, and subsequently filtered using a 0.22-μm filter (Millipore, United States). The cell-free filtrates were concentrated by acetone precipitation (1:4 v/v), and the precipitates were re-dissolved in 100 μl of PBS. An Impranil suspension (100 g l^−1^) in PBS was used as the stock solution for this assay. Dilutions of the Impranil suspension with distilled water were measured to construct a standard curve for converting the absorbance to % clearance. Cell-free precipitates (100 μl) and purified lipase (50 U) were added to 5 ml of the Impranil suspension and incubated at 30°C. The clearance of the Impranil dispersion was measured using a spectrophotometer (DU 730^®^ Life Science UV/Vis spectrophotometer; Beckman Coulter, Brea, CA, United States) at 600 nm for 72 h at 24 h intervals.

Fourier-transform infrared (FTIR) spectroscopy (VERTEX 80v with HYPERION 2000, Bruker, Hardt, Germany) was conducted to determine the changes in the polymer bond formation of PU. After 72 h of incubation, 1 ml of the Impranil-containing supernatant was transferred into Eppendorf tubes and subsequently lyophilized using a freeze dryer (Alpha 1–4 LD plus, Martin Christ, Osterode, Germany). The spectra of freeze-dried samples were recorded at 4 cm^−1^ resolution and wavenumbers from 500 to 4,000 cm^−1^ at room temperature, including non-treated samples as the negative control. All experiments were performed in triplicates under the same conditions.

### 2.6. Degradation of PU foams

Pre-weighed pieces of PS-PU and PE-PU foams (as described in Section 2.1) were added to baffled Erlenmeyer flasks containing 100 ml of R2A medium. The bacterial suspensions of the mid-log phase were inoculated into each flask and incubated on a rotary shaker (180 rpm) at 30°C for 14 days. A negative control without bacterial inoculation was also maintained under the same conditions. After incubation, the weight loss of each foam sample was calculated. The PS-PU and PE-PU foams were thoroughly washed with distilled water five times to remove the colonized bacteria on the surface and air-dried at 50°C until completely dry. The weight loss percentage was calculated as weight loss (%) = (initial weight - final weight)/initial weight × 100. The experiments were performed in triplicate under the same conditions.

The changes in the surface morphology of PU foams were observed using scanning electron microscopy. The PS-PU and PE-PU foams were thoroughly washed with distilled water to remove impurities on the surface and air-dried at 50°C until completely dry. The samples were then gold-coated with a sputter coater (Q15ORS, Quorum, East Sussex, United Kingdom) and observed using an FEI Quanta 250 FEG (FEI, Hillsboro, OR, United States). Non inoculated PU foams were examined as negative controls.

### 2.7. Statistical analyses

One-way ANOVA was performed using SPSS software (version 24, SPSS, Inc., Chicago, IL, United States). The mean values was compared using Scheffé’s method, and *p* values <0.05 were considered statistically significant.

## 3. Results

### 3.1. Isolation and identification of Impranil-degrading strain

**Figure 1:**
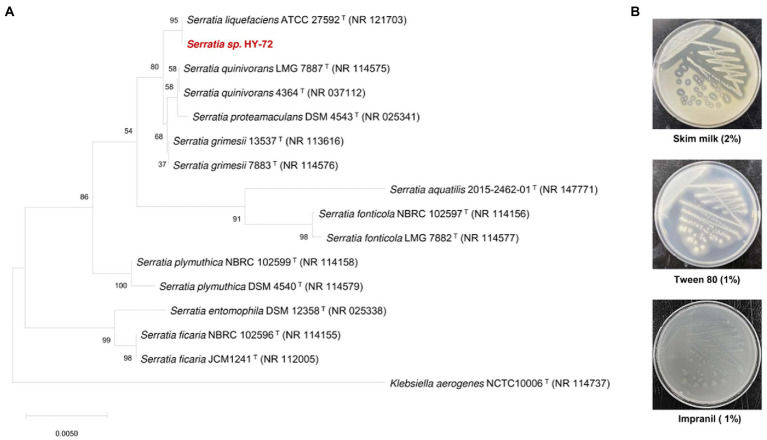
Characterization of HY-72 strain isolated from the intestine of Asian mantis (*Hierodula patellifera*). **(A)** Phylogenetic relationship of the strain HY-72 based on 16S rRNA gene sequence. Neighbor-joining phylogenetic tree based on 16S rRNA gene sequences and closely related species constructed using MEGA X software. Numbers at each branches indicate the bootstrap percentage of 1,000 replications. **(B)** Extracellular enzymatic activities of HY-72 strain.

The Impranil-degrading bacterium with a high degree of transparent halo zones on R2A agar containing 1% (v/v) Impranil was isolated from the intestines of the adult Asian mantis (*H*. *patellifera*). The isolate was selected as a promising candidate for the degradation of PU and was stored for further biodegradation studies. For phylogenetic profiling, the 16S rRNA gene sequence of the isolate (2,000 bp) was compared with those of type strains available in the EzBioCloud database. The isolated strain was most closely related to *Serratia liquefaciens* strain ATCC 27592^T^ (NR121703), with 99.93% 16S rRNA nucleotide sequence similarity (query coverage of 92%; Figure 1A). The HY-72 strain was evaluated for the production of extracellular enzymes associated with hydrolysis of PU components (Figure 1B) The results of agar plate assay indicated that strain HY-72 has the ability to produce extracellular protease (the formation of transparent halo zone) and lipase (the formation of calcium complex); therefore, the isolate has the potential to degrade PU.

### 3.2. Identification and characterization of PU-degrading enzymes

**Figure 2:**
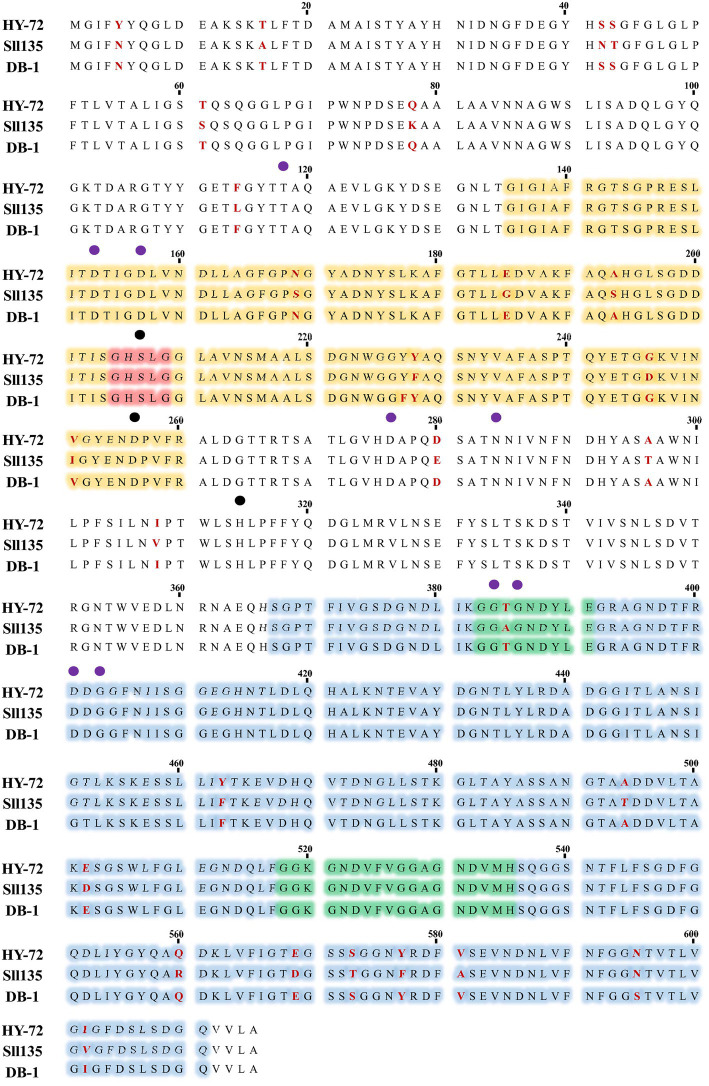
Amino acid sequences of lipase from HY-72 strain. HY-72 lipase, *Serratia liquefaciens* L135 polyurethanase (Sll135; WP_048762691), and *S*. *liquefaciens* S33 DB-1 lipase (DB-1; ABP04234) were compared using CLUSTALW program. Orange = catalytic domain; red; nucleophilic elbow, GXSXG; blue = domain of interaction with calcium; green = glycine-rich repeats, GGXGXDXXX, interacting with calcium and type I secretion system; purple closed circles = amino acid residues that interact with calcium; black closed circles = catalytic triad.

**Figure 3:**
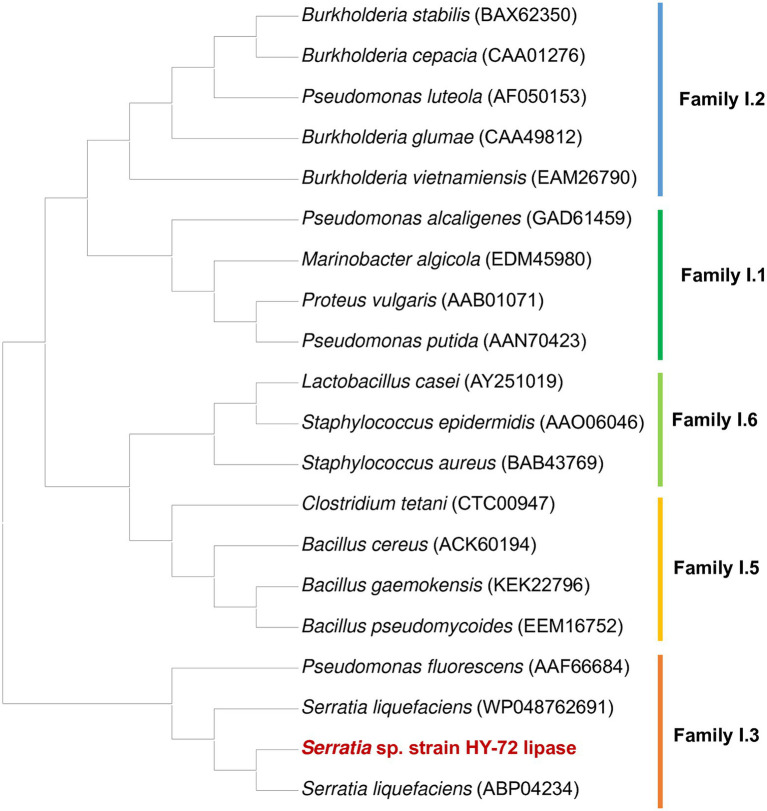
Phylogenetic analysis of HY-72 family I.3 lipase and its closely related functional homologs. Multiple alignments of the amino acid sequences were achieved using Clustal W in the MEGA X software. The protein sequence data used for phylogenetic analysis were retrieved from the GenBank database.

**Figure 4:**
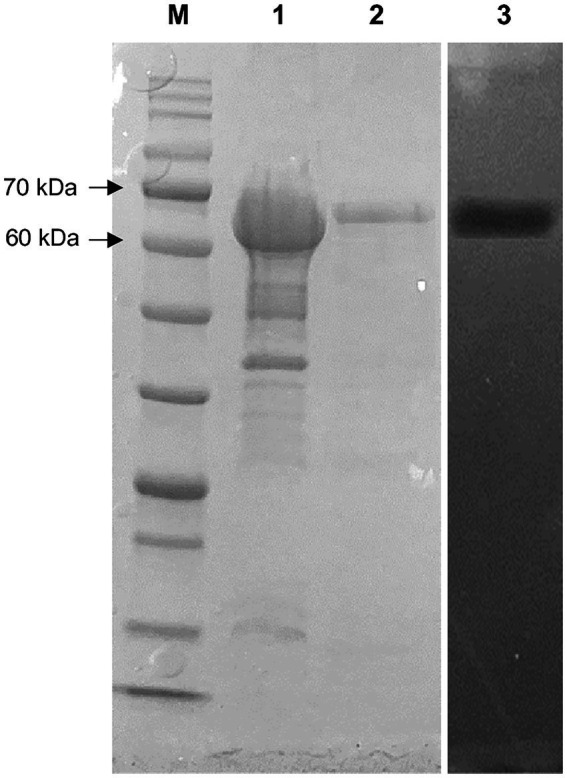
SDS-PAGE and zymography of lipase from HY-72 strain after purification. Lane M, standard marker proteins; lane 1, the total cell lysate of lipase-expressing *Escherichia coli* BL21 after IPTG induction; lane 2, purified lipase; lane 3, corresponding zymogram.

The 1,848 bp lipase gene encoding an extracellular lipase from HY-72 was identified (Figure 2). The nucleotide sequence of the lipase was predicted to express a premature protein of 615 amino acids with a molecular weight of approximately 65 kDa. The active site of the extracellular lipase (serine hydrolase motif), G-X-S-X-G, was found at Gly 205, and the secretion signal, G-G-X-G-X-D-X-X-X, was found at Gly 382. A protein BLAST survey indicated that the primary sequence of premature lipase was similar to that of a *S*. *liquefaciens* S33 DB-1 lipase (ABP04234) with 99.35% identity and *S*. *liquefaciens* polyurethanase (WP_048762691) with 95.77% identity. Phylogenetic analysis showed that the primary sequence of the lipase from HY-72 shared a close evolutionary relationship with that of family I.3 extracellular lipases (Figure 3). The purified recombinant lipase from HY-72 was observed by SDS-PAGE, and their PU degradation activity was confirmed by zymogram analysis. The purified recombinant lipase had a relative molecular mass of approximately 65 kDa, and the gel used to obtain a zymogram revealed PU-clear regions (Figure 4). The specific lipolytic activity of the recombinant PU-degrading lipase for *p*NPA was 2,883 U mg^−1^ (Table 1).

**Table 1:** Enzyme characteristics of lipase for polyurethane degradation.

Strain	M*r* (kDa)	Optimal pH	Optimal Temp. (°C)	Specific activity (U mg^−1^)	Reference
*Serratia* sp. HY-72	65	8.0	30	2,883^a^	This study
*Serratia liquefaciens* L135	65	8.0	30	2,793^b^	Salgado et al., 2021
*Acinetobacter gerneri* P7	66	8.0	25	37.58^c^	Howard et al., 2012
*Pseudomonas chlororaphis*	63	8.4	-	8.5^a^	Ruiz et al., 1999

One unit (U) of lipolytic activity is defined as the amount of protein that hydrolyzed 1 μmol p-nitrophenol ml^−1^ min^−1^. All assays were performed in triplicates.

a Specific activity toward p-nitrophenyl acetate.

b Specific activity toward p-nitrophenyl palmitate.

c Specific activity toward p-nitrophenyl propanate.

### 3.3. Biodegradation of Impranil

**Figure 5:**
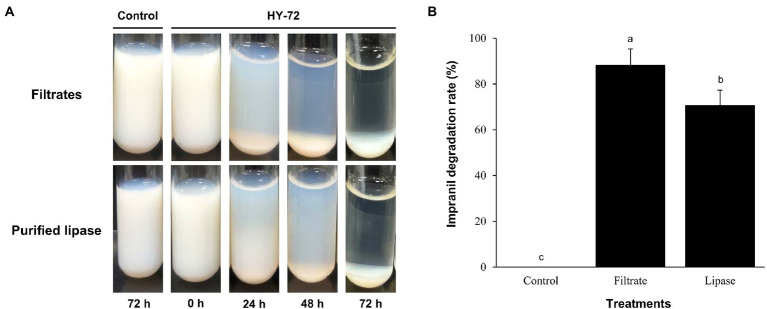
Time course of degradation of Impranil (100 g l^−1^) by the cell-free filtrates and purified lipase of HY-72 strain. **(A)** Photograph of Impranil suspensions according to incubation with the cell-free precipitates and purified lipase of HY-72 strain compared to the non-treated control. **(B)** Quantitative analysis of Impranil degradation. Percent clearance was determined by a spectrophotometer at 600 nm. All data were normalized to the negative control. Values followed data are presented as means ± SD (*n* = 3). Different letters above error bars indicate a significant difference by Scheffé’s test (*p* < 0.05).

**Figure 6:**
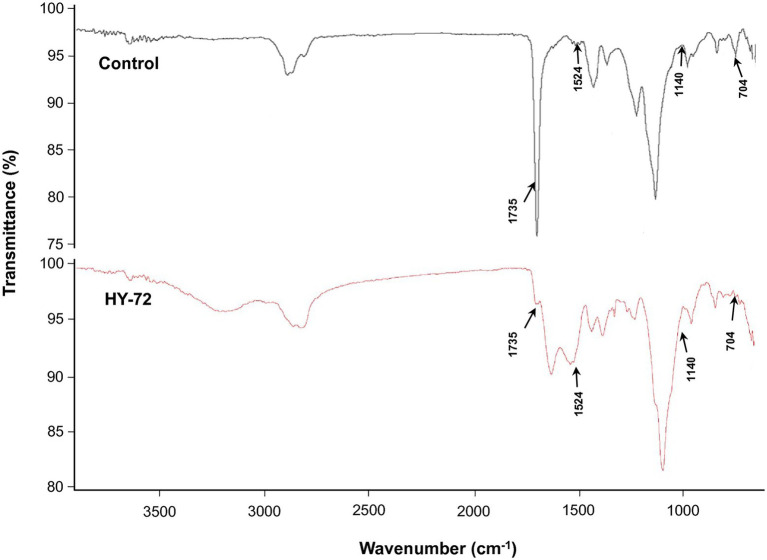
Comparison of FTIR spectra of Impranil degradation after 3 days of incubation with the cell-free precipitates and purified lipase of HY-72 strain and non-treated control. Arrows represent the position of peaks for the functional groups decreased or increased in intensity.

The strain HY-72 and lipase were quantitatively evaluated by turbidimetric analysis to confirm the biodegradation of Impranil. Optical absorbance at 600 nm was used as a direct measurement of clearance by the bacterial filtrates and purified lipase. FTIR analysis was performed to confirm Impranil biodegradation. Visual observation of the Impranil suspension treated with filtrates of HY-72 and purified lipase showed that the turbid suspension became transparent and the value of OD_600_ notably decreased in a time-dependent manner (Figure 5A). After 72 h incubation, the degradation rate of the bacterial filtrates and the purified lipase was 85.24 and 70.37%, respectively, while the negative control not treated with HY 72 did not show detectable clearance of the suspension (Figure 5B). FTIR spectra of Impranil degradation by the strain HY-72 and its lipase revealed changes in functional groups, accompanied by more subtle changes at other wave numbers (Figure 6). The filtrates and purified lipase from HY-72 demonstrated a significant decrease in the signal intensity of the peak at 1,735 cm^−1^, indicating that the ester functional group was affected by the hydrolysis and subsequent catabolism of urethanes. The peak at 1,524 cm^−1^ corresponds to the N-H bending, with a notably increase in the C-N stretch. The characteristic peaks from the urethane group at 1,140 cm^−1^, representing C-O stretching, also increased in intensity. The peak at 704 cm^−1^ (aliphatic chain signals) decreased after 72 h of incubation. The results correlated with the biodegradative enzymatic activities detected in the HY-72 filtrates, as indicated by the degradation assays and FTIR analysis.

### 3.4. Biodegradation of PS and PE-PU foam

**Figure 7:**
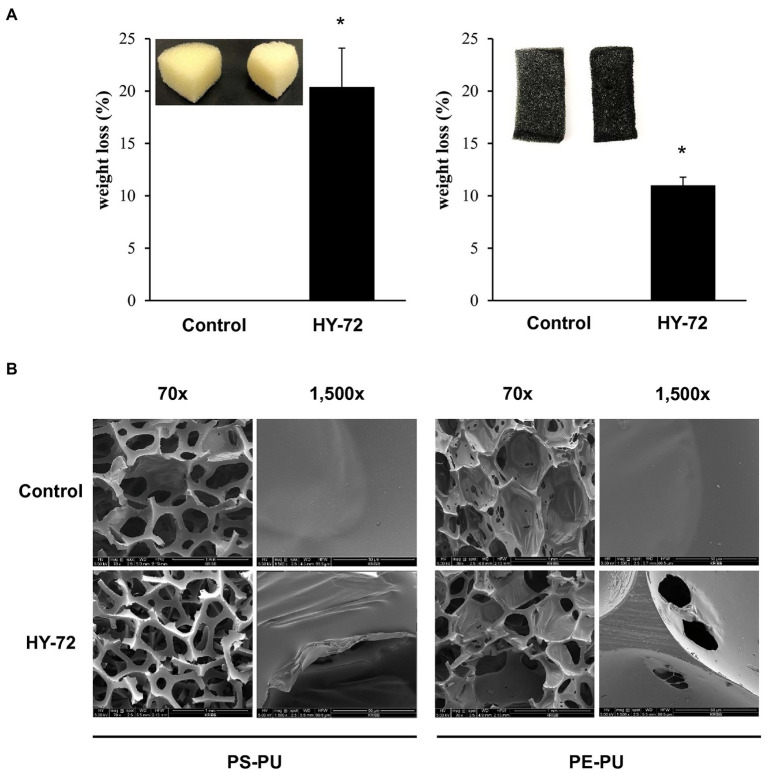
Weight loss **(A)** and scanning electron micrographs **(B)** of PU foam by HY-72 strain. PS-PU foam and PE-PU foam were incubated with the HY-72 strain for 14 days. Each pair of foam pieces presents the control foam (left) compared to the HY-72 treated foam (right). Values followed data are presented as means ± SD (*n* = 3). Statistical significance between compared groups is indicated as * *p* < 0.05.

The degradation efficiency of the strain HY-72 for two types of PU foams (PS-PU and PE-PU) was determined by weight loss measurement and analyzing scanning electron micrographs. PS-PU and PE-PU foams showed approximately 20.34 and 5.13% weight loss, respectively (Figure 7A). The dry weight of untreated foams did not change after 2 weeks of incubation. The PU foams incubated with the bacterial isolate showed morphological changes caused by bacterial degradation. In scanning electron micrographs, PS-PU foam treated with the strain HY-72 showed holes and loss of integrity of their reticulated cell structure in panoramic views (70 × magnification); the development of bends was detected in a closer view (1,500 × magnification). Similarly, the PE-PU foam structure was disrupted, and the development of cracks, roughness, and surface roughening was evident, while the non-treated control foam maintained its structure and a smooth, intact and clear surface (Figure 7B).

## 4. Discussion

Insects exhibit remarkable adaptations to various environmental factors. These adaptation are closely related to the changes in the profile of their gut microorganisms as well as associated functions (Dillon and Dillon, 2004). Insect-associated microbes produce various bioactive molecules and effective digestive enzymes, which have great potential for industrial applications (Jang and Kikuchi, 2020). Insect-associated gut microbes have been studied for their bioremediation ability using contaminants, pollutants, and toxins as nutritional sources (Loredo-Treviño et al., 2012; Banerjee et al., 2021). Insects and their gut microbes have gained attention as promising resources for plastic biodegradation (Bilal et al., 2021). Recent studies have reported that bacterial strains from *Tenebrio molitor* (Yang et al., 2015) and *Triboluim castaneum* (Wang et al., 2020) efficiently degrade polystyrene. Bacteria isolated from *Plodia initerpunctella* (Yang et al., 2014) and *T*. *molitor* (Yin et al., 2020) have been reported to degrade PE. This study evaluated insect symbiotic microbes and their secreted lipases for PU biodegradation. *Serratia* sp. strain HY-72 was isolated from the intestine of the adult Asian mantis (*H*. *patellifera*), and its PU-degradation activity was investigated. This is the first report demonstrating PU degradation by a *Serratia* sp. and its exogenous lipases isolated from the intestine of the insects.

Various bacteria utilize exogenous enzymes to degrade polymers, and use them as nutritional sources for their assimilation and metabolism. Several extracellular enzymes responsible for PU biodegradation have been studied (Biffinger et al., 2015). The enzymes that degrade PU by hydrolysis include protease, lipase, esterase, and urease, and the synergistic action of these enzymes could improve the biodegradation of PU through their co-metabolic interactions (Howard, 2002; shah et al., 2016). Based on the results, it was hypothesized that the biodegradation of PU by HY-72 strain mainly occurs *via* extracellular enzymatic activities. The genus *Serratia* produces exogenous enzymes, such as proteases and esterases (Gupta et al., 2004). The 65 kDa purified recombinant lipolytic enzyme from HY-72 showed lipase activity and degraded PU. The results were consistent with those of a previous study in which 65 kDa lipase from *S*. *liquefaciens* L135 from cow’s milk had lipolytic activity, and the modeled and validated structure of this polyurethanase was able to bind urethane by molecular docking (Salgado et al., 2021). Comparing the purified recombinant lipase from HY-72 with polyurethanase from *S*. *liquefaciens* L135, there were 26 amino acids, of which 6 were different in the catalytic domain and 10 in the interaction domain with calcium. In addition, Thr 385 of the secession signal in the interaction domain with calcium was different. The specific activity of lipase from HY-72 was 2,883 U mg^−1^, and polyurethanase from *S*. *liquefaciens* L135 was 2,793 U mg^−1^, using *p*NPA as a substrate (Salgado et al., 2021). The present study confirms family I.3 lipase has a PU-degrading ability.

Impranil has been widely used as a substrate for detecting the PS-PU hydrolyzing activity of bacterial strains (Schmidt et al., 2017); it is an aqueous aliphatic polymer composed of spheres 200 nm or less that can remain suspended in aqueous media (Biffinger et al., 2015). The HY-72 filtrates and purified recombinant lipase showed PU degradation rates of 85.24 and 70.37%, respectively. Various types of protease are known to hydrolyze urethane bonds, leading to release of carbon dioxide and formation of amines and alcohols (Mahajan and Gupta, 2015). A previous study suggested that all tested strains with PU degradative capabilities were closely related in their protease activity (Loredo-Treviño et al., 2012). In this study, the higher PU degradation rate achieved by cell filtrates is believed to be due to a synergistic effect between the protease and lipase of the HY-72 strain. In comparison, *Pseudomonas putida* A12 from soil showed that Impranil was degraded by 45% after 2 days (Peng et al., 2014). *Lasiodiplodia* sp. strain E2611A and *Cladosporium pseudocladosporioides* T1.PL.1 showed 85 and 87% Impranil degradation rate after 14 days (Russell et al., 2011; Álvarez-Barragán et al., 2016). The FTIR spectrum analysis demonstrated Impranil breakdown due to the attack on functional groups, in accordance with those previously reported by Peng et al. (2014) and Álvarez-Barragán et al. (2016). Based on the Impranil degradation assay and FTIR analysis, the strain HY-72 and family I.3 lipase could be a potential resource for the biodegradation of colloidal PS-PU. The quantitative and visual analyses of PS- and PE- PU foam degraded by the strain HY-72 indicated that PS-PU (with weight loss of 20.34%) had a higher degradation rate than PE-PU after 2 weeks of incubation. These results indicate that the strain HY-72 and its exogenous enzyme could also participate in the biodegradation of foam type-PU, which has urethane and ester groups. In the scanning electron micrographs, the PS- and PE-PU foams showed disruption and loss of integrity of their reticulated cell structure and changes in the surface morphology, which is consistent with previous reports (Gautam et al., 2007; Spontón et al., 2013; Gómez et al., 2014; Pellizzi et al., 2014; Álvarez-Barragán et al., 2016; Pérez-Lara et al., 2016; Khan et al., 2020). Therefore, the strain HY-72 has the potential to biodegrade PU. However, practical biodegradation on a large scale and information on the bioconversion of PU by microbial strains are limited and remains a challenging subject for managing plastic waste. Therefore, further studies are needed to verify the metabolic pathways and the mechanism of bioconversion, also its degradation system optimization.

**Figure 8:**
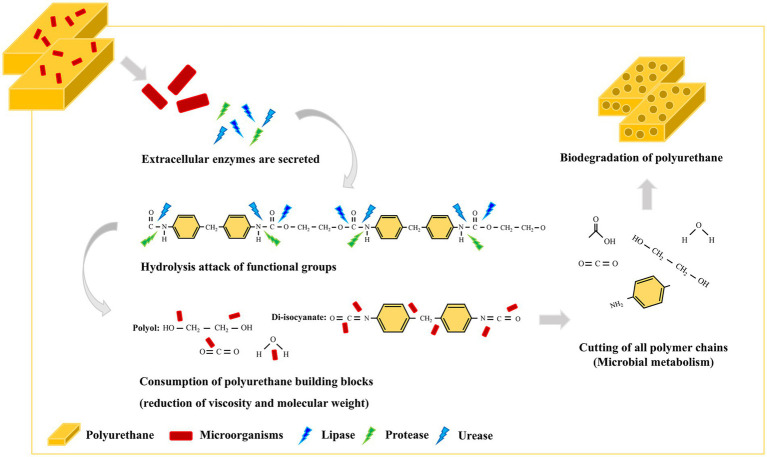
Schematic illustration of the degradation of polyurethane by the HY-72 strain.

A PU-degrading bacterium, *Serratia* sp. strain HY-72, was isolated from the intestine of the Asian mantis (*H*. *patellifera*) and evaluated its PU-degrading activity. The results confirmed that this strain can degrade aqueous aliphatic PS-PU polymer, Impranil, and two types of foam (PS- and PE-PU; Figure 8). The 65 kDa lipase of I.3 lipase family isolated from this strain was identified as a PU-degrading enzyme with high specific lipolytic activity. Further studies on the degradation mechanism and its optimization as well as on the practical applications of the HY-72 strain for real-world PU waste management is required and should employ multi-omics (e.g., genomics, proteomics, metabolomics), synthetic microbial communities (e.g., cell–cell interaction, microbial consortium, molecular interaction), and gene editing tools (CRISPR, TALEN, and ZFN; Jaiswal et al., 2020; Liu et al., 2021). The present study suggests that the insect-associated bacteria and their exogenous enzymes have potential applications in plastic biodegradation, and can offer a promising approach for plastic waste management.

## Data availability statement

The datasets presented in this study can be found in online repositories. The names of the repository/repositories and accession number(s) can be found in the article/supplementary material.

## Author contributions

J-HK, K-HS, and H-YP participated in acquiring the data and the study design, drafted the manuscript, and revised the final manuscript. SHC performed the experiments and analyzed the data. MGP and DHP participated in study protocol design and supplied samples. All authors contributed to the article and approved the submitted version.

## Funding

This research was supported by the Korea Research Institute of Bioscience and Biotechnology (KRIBB) Research Initiative Program (KGM5492221).

## Conflict of interest

The authors declare that the research was conducted in the absence of any commercial or financial relationships that could be construed as a potential conflict of interest.

## Publisher’s note

All claims expressed in this article are solely those of the authors and do not necessarily represent those of their affiliated organizations, or those of the publisher, the editors and the reviewers. Any product that may be evaluated in this article, or claim that may be made by its manufacturer, is not guaranteed or endorsed by the publisher.

## Footnotes

^1^ http://www.cbs.dtu.dk/services/SignalP/

^2^ http://pfam.sanger.ac.uk/

^3^ http://www.ncbi.nlm.nih.gov/BLAST/
